# Does malalignment affect revision rate in total knee replacements: a systematic review of the literature

**DOI:** 10.1186/s40064-015-1604-4

**Published:** 2015-12-30

**Authors:** Mohammed Hadi, Tim Barlow, Imran Ahmed, Mark Dunbar, Peter McCulloch, Damian Griffin

**Affiliations:** Trauma and Orthopaedic Department, UHCW, University of Warwick, Coventry, CV2 2DX UK; John Radcliffe Hospital, University of Oxford, Oxford, OX3 9DU UK

**Keywords:** Malalignment, Total knee arthroplasty, Revision rate, Systematic review

## Abstract

To ensure implant durability following Modern total knee replacement (TKR) surgery, one long held principle in condylar total knee arthroplasty is positioning the components in alignment with the mechanical axis and restoring the overall limb alignment to 180° ± 3°. However, this view has been challenged recently. Given the high number of TKR performed, clarity on this integral aspect of the procedure is necessary. To investigate the association between malalignment following primary TKR and revision rates. A systematic review of the literature was conducted using a computerised literature search of Medline, CINHAL, and EMBASE to identify English-language studies published from 2000 through to 2014. Studies with adequate information on the correlation between malalignment and revision rate with a minimum follow-up of 6 months were considered for inclusion. A study protocol, including the detailed search strategy was published on the PROSPERO database for systematic reviews. From an initial 2107 citations, eight studies, with variable methodological qualities, were eligible for inclusion. Collectively, nine parameters of alignment were studied, and 20 assessments were made between an alignment parameter and revision rate. Four out of eight studies demonstrated an association between a malalignment parameter and increased revision rates. In the coronal plane, only three studies assessed the mechanical axis. None of these studies found an association with revision rates, whereas four of the five studies investigating the anatomical axis found an association between malalignment and increased revision rate. This study demonstrates the effect of malalignment on revision rates is likely to be modest. Interestingly, studies that used mechanical alignment in the coronal plane demonstrated no association with revision rates. This questions the premise of patient specific instrumentation devices based on the mechanically aligned knee when considering revision as the endpoint.

## Background

Modern total knee replacement (TKR) is considered an effective treatment for knee arthritis (Callahan et al. 1994). Over 77,000 TKR operations were performed during 2013 in England and Wales (Registry 2013) with expectations of increasing demand (Kane Rl Sk and Al 2003). To ensure implant durability, one long held principle is positioning the components in alignment with the mechanical axes and restoring the overall limb alignment to 180° ± 3° (Jeffery et al. 1991; Lotke and Ecker 1977). In vitro studies using simulators (D’lima et al. 2001), finite model analysis (Cheng et al. 2003), and cadaveric studies (Green et al. 2002), have backed this notion. This resulted in a substantial investment in means such as computer-assisted technologies to achieve better alignment outcomes (Mason et al. 2007; Siston et al. 2007). Numerous investigators asserted the importance of alignment to avoid poor outcomes following TKR, in particular implant failures requiring revision surgery (Bargren et al. 1983; Longstaff et al. 2009; Lotke and Ecker 1977; Moreland 1988; Nicoll and Rowley 2010; Ritter et al. 1994; Tew and Waugh 1985; Werner et al. 2005).

Recently, reasons to challenge this view have emerged. It is suggested that the evidence of poor outcomes secondary to malalignment is largely historic and based on studies of inferior implant designs, some of which have been discontinued (Bach et al. 2009; Bonner et al. 2011; Matziolis et al. 2010; Parratte et al. 2010), and the use of poor radiological assessment techniques to assess for malalignment (Lotke and Ecker 1977). Outcomes following computer assisted TKR, proven to achieve better target alignment in comparison to conventional techniques, have demonstrated little evidence of long term clinical advantage (Cheng et al. 2012; Matziolis et al. 2010).

The choice of target for ideal mechanical alignment has been challenged by proponents of kinematically aligned TKR who have reported promising results (Howell et al.  2013a, b). Kinematic alignment aims to place the femoral component so that its transverse axis coincides with the primary transverse axis in the femur about which the tibia flexes and extends. With the removal of osteophytes the original ligament balance can be restored and the tibial component is placed with a longitudinal axis perpendicular to the transverse axis in the femur. Contrast this to conventional and computer assisted mechanically aligned techniques which aim to place the femoral component perpendicular to the mechanical axis of the femur, the tibial component perpendicular to the mechanical axis of the tibia and to rotate the femoral component so that flexion and extension gaps are parallel. As a result a mechanical malalignment (where the components are not positioned at 180° ± 3°) will differ for a kinematically aligned knee where the planned implant alignment is outside the 180° ± 3° range.

Radiological assessment of malalignment is based on how close to the mechanical axis the prostheses have been implanted on different planes. In the literature, there is a lack of consistency in assessing and subsequently describing malalignment (Kamath et al. 2010). For example, the coronal TKR alignment can be measured in relation to the hip-knee-ankle axis (limb mechanical axis) on images of the whole limb, or relative to the femoral and tibial intramedullary anatomical axes on short knee films. The same applies to sagittal and axial assessments. Short leg anatomical axes are usually converted to an approximation of the mechanical axis, although this process is prone to error.

The aim of this study is to explore the recent evidence on the effect of malalignment on TKR longevity. We set out to answer the following research question: In patients undergoing primary TKR, is malalignment associated with increased revision rates?

## Methods

This review followed the guidelines described by the agency for healthcare research and quality (AHRQ) criteria (Viswanathan et al. 2008). The review has been registered and a protocol has been published on the PROSPERO database; protocol number 2012:CRD42012001914 (Mohammed Hadi Md and Barlow 2012).

### Literature search

A computerised literature search of the following databases was carried out: (MEDLINE), (CINHAL), (EMBASE). A broad search strategy was adopted. The aim was to identify all English-language studies published from 2000 through to 2014 in order to assess data related to current implant designs. The last search was performed on September 2014. A manual search of bibliographies of all eligible and other relevant publications was also undertaken.

### Eligibility criteria

Both observational and experimental designs were considered.

#### Inclusion criteria

- All patients eligible for a primary TKR.
- All open procedures that used a total condylar implants.
- All described approaches.
- All radiological alignment assessment methods and parameters described.

#### Exclusion criteria

- Studies without adequate or clear information on the correlation analysis between malalignment and revision rate.
- Studies with a mean follow-up of less than 6 months.
- Abstract-only publications, expert opinions and chapters from books.

### Extraction of data

Two investigators (MH, TB) independently reviewed the titles and abstracts to identify and retrieve all relevant articles and performed the data extraction. Any disagreement was settled by consensus between the two reviewers or with a third investigator (MD).

### Quality assessments of included studies

All studies were assessed for their methodological qualities in accordance with their study design. Case control and Cohort studies were assessed using the Ottawa-Newcastle score star system (Stang 2010). Case series were assessed using an AHRQ design-specific scale (Viswanathan et al. 2008).

**Fig. 1:**
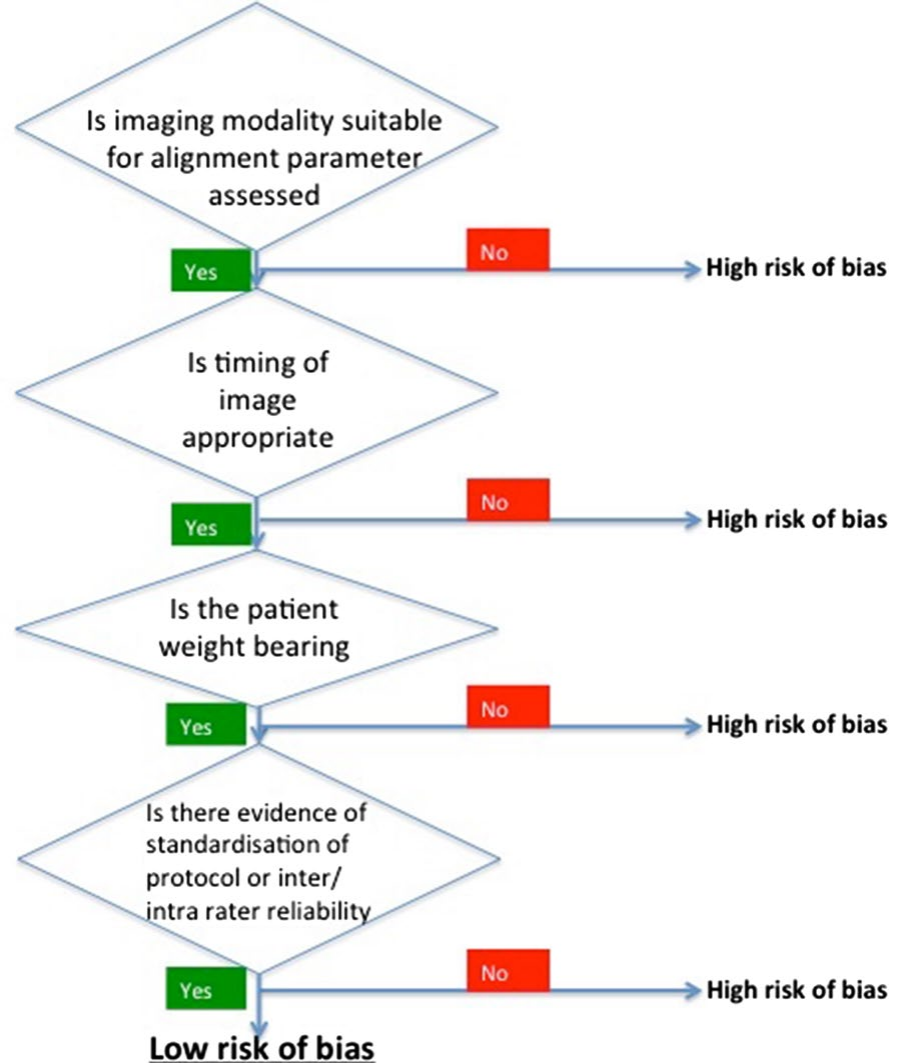
Radiological assessment quality (RAQ) criteria for assessing alignment. The evaluation was done using a five yes/no question checklist that was devised for this review. A sensitivity analysis was performed to determine if the quality of the radiological methods was an important factor in the outcome. The rationale for each set of questions was as follows: The suitability of the imaging modality used: Overall limb alignment is better assessed on a whole leg radiograph compared to a short film radiographs (Moreland 1988) and Short film x-rays are used for the assessment of component’s anatomical alignment (Morgan et al.  2008). The timing of the imaging: Malalignment on images acquired several years following surgery may be secondary to implant subsidence/migration (Morgan et al.  2008). The patient’s weight bearing status at the time of imaging: the relationship between the bony and soft tissue parts of the knee joint is most visible during stressing manoeuvre such as weight bearing (Nicoll and Rowley 2010). Indication of standardisation when acquiring the images: Non-standardised protocols for acquiring images can result in inconsistent magnification and rotation, introducing a source of bias (Parratte et al. 2010; Registry 2013). Evidence of rater reliability when assessing the images for alignment: To ensure consistency (Parratte et al. 2010; Registry 2013)

Studies were further evaluated based on the quality of their radiological methods for assessing alignment. The evaluation was done using a five-question checklist devised for this review; the Radiological Assessment Quality (RAQ) criteria. The items in the checklist, together with their corresponding justification, are described in Fig. 1. Studies were deemed as low, unclear or high risk of assessment bias based on the radiological methods described. (Berend et al. 2008; Bhandari et al. 2013; Cooke and Sled 2009; Hirschmann et al. 2011; Leach et al. 1970; Scuderi et al. 2012).

### Statistical analysis

Due to the exploratory nature of the research question, the summary of data was focused on descriptive statistics and qualitative assessment of the content of the identified literature. Formal meta-analysis not conducted due to the variety of measures of alignment, and the varying methodological quality of the studies. Meta-analysis could cloud the picture by producing a precise, but potentially spurious result, rather than provide an adequate summary.

## Results

**Fig. 2:**
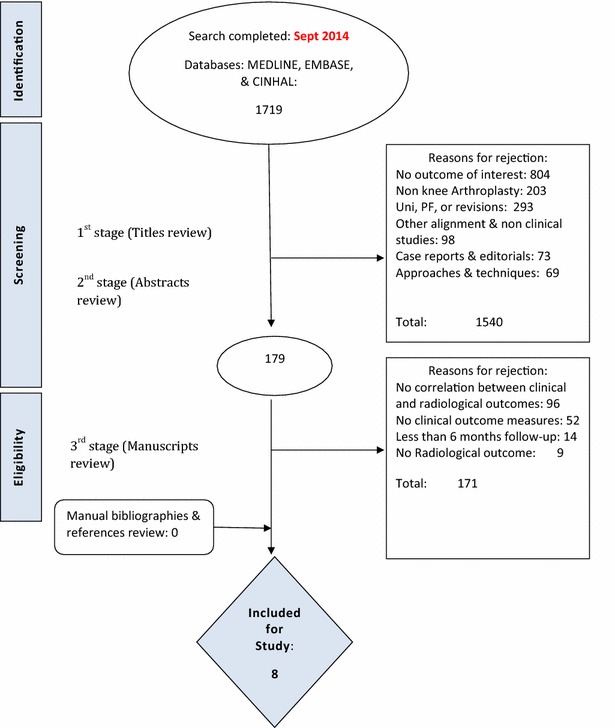
PRISMA flow diagram including the details of our search results for this review. Figure shows the reasons behind study exclusion at each stage of the search and the number of studies identified at each point of the search

The initial search returned 2107 citations, of which 1719 were considered for screening. 179 studies were selected for manuscript review stage. Most studies were excluded at the title and abstract screening stage (n = 1540); the main two reasons for exclusions were duplication and the lack of outcome of interest. Details of the study selection process are described in Fig. 2.

A total of eight studies (Berend et al. 2004; Bonner et al. 2011; Fang et al. 2009; Kim et al. 2014; Magnussen et al. 2011; Morgan et al.  2008; Parratte et al. 2010; Ritter et al. 2011) fulfilled the inclusion criteria and were eligible for analysis. No RCTs fulfilled the inclusion criteria. Although many RCTs reported alignment data, none examined the correlation with revision rates. All studies were from single centres apart from one (Kim et al. 2014), four studies were from North America (Berend et al. 2004; Fang et al. 2009; Parratte et al. 2010; Ritter et al. 2011), three studies from Europe (Bonner et al. 2011; Magnussen et al. 2011; Morgan et al.  2008) and one from Asia (Kim et al. 2014). Five studies declared receiving no funds or sponsorship from any commercial or industry related organisation (Bonner et al. 2011; Fang et al. 2009; Kim et al. 2014; Parratte et al. 2010; Ritter et al. 2011). Table 1 demonstrates key study characteristics.

**Table 1:** A table demonstrating key characteristics of the studies selected for review

Author	Study design	Sample size	Follow up (mean range)	Number of patients lost to follow up	Final study sample size	Quality assessment score	Judgement risk on bias
Berend et al. (2004)—CORR	Case series	8598 (5535) from database	5 years 2–14.2 years		3152 (2125). 41 tibial failures were analysed	AHRQ—All four factors were present	Low risk
Fang et al. (2009)—J Arthro	Case series	6070 (3992) from database	6.6 years 2–22.5 years	1118 (28.0 %) patients died	6277	AHRQ—All four factors were present	Low risk
Ritter et al. (2011)—JBJS	Case series	9483	7.6 ± 3.8 years 2–22.5 years	3404	6079	AHRQ—All four factors were present	Low risk
Kim et al. (2014)—international orthopaedics	Case series	3150	15.8 years 11–18 years	102	3048	AHRQ—three out of four factors present	Low risk
Morgan et al. (2008)—international orthopaedics	Case series	197	9 years	No mention	197	AHRQ—two out of four factors present	Unclear/high risk
Parratte et al. (2010)—JBJS	Case control	417	Minimum 15 years	19	398	Ottawa—Newcastle score eight	Low risk
Bonner et al. (2011)—JBJS	Case control	501	9.8 years ?–15 years	184 (died before last review, however, survival data included in analysis)	458	Ottawa—Newcastle score seven	Low risk
Magnussen et al. (2011)—CORR	Case control	608	Median 4.7 years 2–19.8 years	55	553	Ottawa—Newcastle score seven	Low risk

The total number of patients recruited combined in all studies was 20,162 patients. Minimal but comparable patient baseline characteristics were reported.

**Fig. 3:**
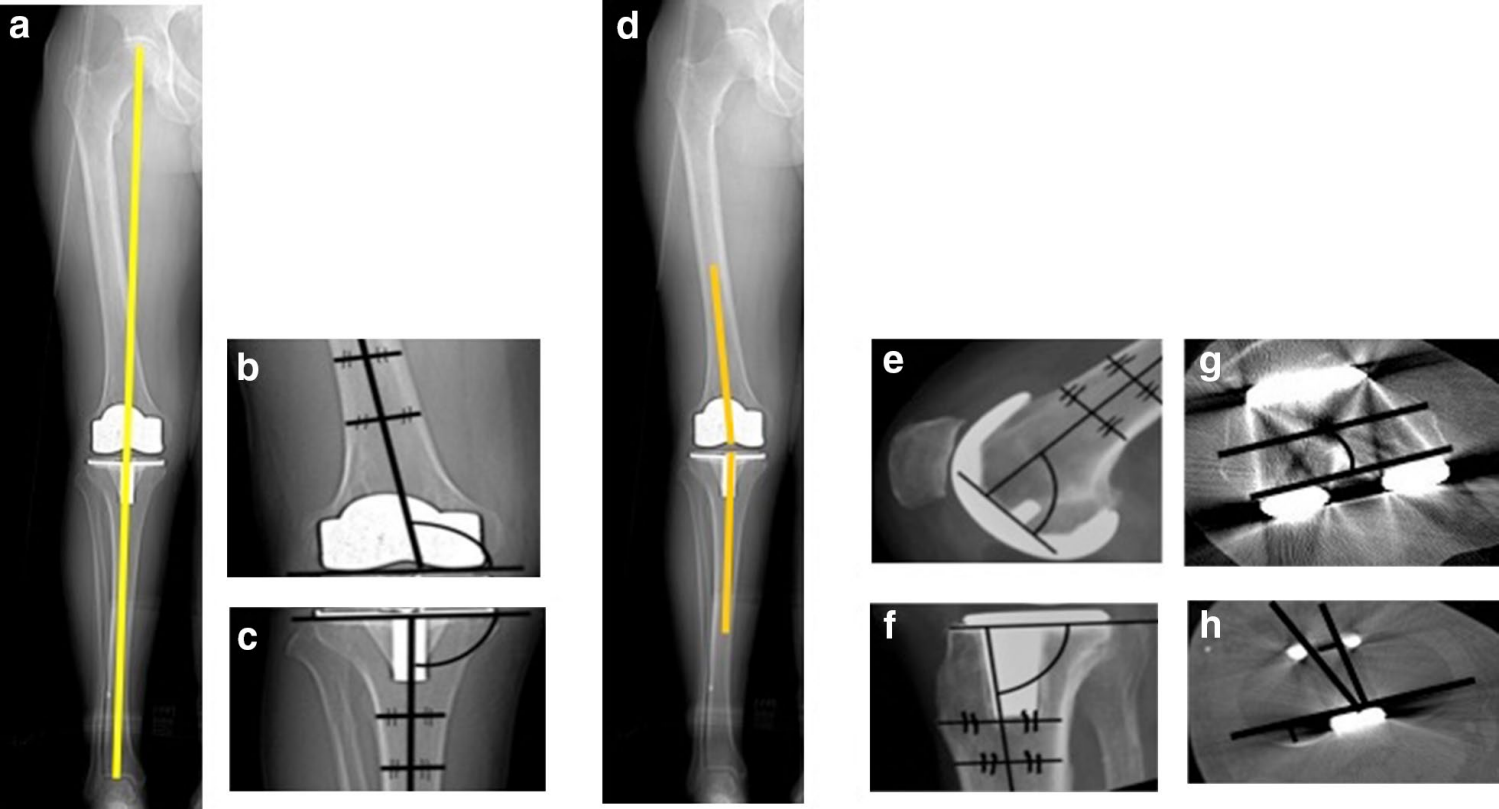
A diagrammatic representation of different alignment parameters based on the knee society total knee arthroplasty roentgenographic evaluation and scoring system24. The coronal tibiofemoral mechanical angle is the angle resulting from drawing a line from the centre of the femoral head down to centre of the ankle through the centre of the knee (**a**)—ideally 180°. The coronal femoral angle cFA (**b**)—ideally 96°—and coronal tibial angle cTA (**c**)—ideally 90°—are the angles between the components’ coronal axes (the line connecting the femoral components most distal condyles and the line along the horizontal tibial plate) and the bones’ coronal anatomical axes (line which bisects the medullary canal of the femur and tibia respectively). The coronal tibiofemoral anatomical angle is a combination of the coronal anatomical femoral axis and coronal anatomical tibial axis (**d**). The sagittal femoral sFA (**e**)—ideally 90°—and sagittal tibial sTA (**f**)— ideally between 83 and 90°—angles are the angles between the components’ sagittal axes (horizontal line perpendicular to the femoral component peg and line along the horizontal tibial plate) and the anatomical sagittal bones’ axes (line which bisects the medullary canal of the femur and tibia respectively). The axial femoral (aFRA) (**g**)—ideally 0°—and axial tibial—ideally within 15°—(aTRA) (**h**) angles are the angles between the components’ axial axes (line through the centre of the femoral pegs and the line through the most posterior points of the tibial plate on axial views respectively) and the bones’ axial axes (surgical epicondylar femoral axis and the tibial tuberosity axis respectively). The combined components axial (aCRA) rotational alignment angles—ideally 0°—is the angle between the components axial axes

The included malalignment parameters are demonstrated in Fig. 3; these are:

- Coronal malalignment: Malalignment of the components relative to the limb mechanical axis. This relation was presented using different parameters including the coronal tibio-femoral mechanical angle (cTFmA); the hip-knee-ankle angle, the coronal tibio-femoral anatomical angle (cTFaA) which is the angle between the femoral and tibial anatomical intramedullary long bone axes, and the coronal femoral angle (cFA) and the coronal tibial angle (cTA) which are the angle between the component axes and the anatomical intramedullary long bone axes.
- Sagittal malalignment: Malalignment of the components relative to the limb intramedullary long bone sagittal axis; the sagittal femoral (sFA) and tibial (sTA) angles.
- Axial malalignment: the axial femoral (aFRA) and the axial tibial (aTRA) angles which represent the component malalignment relative to the surgical epicondylar axis and axial tibial rotational axis respectively. And the combined components axial (aCRA) rotational alignment angle.

### Methodological qualities assessment

The methodological quality assessment is presented in Table 1.

### Radiological qualities assessment

Varying radiological assessment methods were used amongst include studies. The radiological quality assessment of included studies is presented in Table 2.

**Table 2:** Studies radiological methods quality assessment

	Modality of imaging	Timing of imaging	Weight bearing	Protocol/standardisation	Rater reliability assessment	Outcome
Berend et al. (2004)	SLR	At follow up	Y	U	N	High risk
Bonner et al. (2011)	LLR	6 months	Y	Standardised	N	Low risk
Fang et al. (2009)	SLR	Varied	Y	Y	N	High risk
Kim et al. (2014)	CT, LLR	1 week	Y	Y	Y	Low risk
Magnussen et al. (2011)	LLR	Follow up	Y	YRoutine for Database	Y	Low risk
Morgan et al. (2008)	LLR	Immediate post op	Y	Y	N	low risk
Parratte et al. (2010)	LLR	2–3 month post op	Y	YStandardised protocol	Y	Low risk
Ritter et al. (2011)	SLR	Latest follow up	*Y*	U	N	High Risk

Assessment of radiological methods used to assess alignment for this review. We devised a five point checklist (Fig. 1) and all studies were assessed using this checklist to identify whether they were high/low risk. *CT* computerised tomography, *LLR* Long leg radiograph, *SLR* Short leg radiograph, *Y* yes, *N* No, *U* Unknown

#### Association between malalignment and revision rate

### Mechanical alignment

Three studies (Bonner et al. 2011; Magnussen et al. 2011; Parratte et al. 2010) assessed malaligment relative to the mechanical axis. These studies were at low risk of radiological bias, and low risk of methodological bias as judged by the RAQ criteria and the quality assessment criteria. These studies reported no significant association between malalignment and increased revision rates (Table 3).

**Table 3:** Tibio-femoral mechanical angle malalignment (cTFmA)

Author	RAQ criteria for radiological bias	Association between malalignment and worse outcome	Sample size	Alignment data	Findings
Parratte et al. (2010)	Low risk	No	398	292 knees classed as mechanically aligned 0° ± 3. 10 knees in the outlier group (beyond 0° ÷ 3°	15.4 % revision rate in the mechanically aligned group. 13 % in the outlier group (p = 0.88). No association between malalignment and revision
Bonner et al. (2011)	Low risk	No	458	372 knees were classified as mechanically aligned (0° ± 3°). 86 knees were within the malaligned group	33 revisions for aseptic loosening. Kaplan–Meier survival analysis showed a weak tendency towards improved survival with restoration of a neutral mechanical axis, *but this did not reach statistical significance (p* *=* *0.47)*
Magnussen et al. (2011)	Low risk	No	553	181 patients were in varus alignment, 352 were in neutral alignment and 20 were in valgus alignment	No *statistically significant difference* in revision rates between the three groups (p = 0.15)

### Anatomical alignment

Malalignment on the coronal plane was associated with worse revision rates in a total of four studies, all of which used anatomical axes to measure malalignment (Berend et al. 2004; Fang et al. 2009; Kim et al. 2014; Morgan et al.  2008; Ritter et al. 2011). When each component was analysed in turn, femoral malalignment was associated with increased revision rates in two studies (50 %), and tibial malalignment in three studies (75 %). Details of coronal malalignment and revision rates for each measure of all studies is presented in (Table 4).

**Table 4:** Studies investigating association between coronal malalignment and revision rates

Author	RAQ criteria for radiological bias	Association between malalignment and worse outcome	Sample size	Alignment data	Findings
Tibial angle malalignment (cTA)					
Berend et al. (2004)	High risk	Yes	3152	376 knees had >3° varus alignment	21 revisions due to medial bone collapse all in relative varus. Tibial component with >3° varus had *increased risk of failure p* *<* *0.0001*
Fang et al. (2009)	High risk	Yes	6277	Individual tibial alignment figures not stated	2.8 times increased risk of failure by medial tibial collapse *p* *=* *0.04*
Ritter et al. (2011)	High risk	Yes *(non significant)*	6079	81.9 % knees defined as neutral. Neutral defined as ≥90°	3.2 % failure rate *p* *=* *0.492*
Kim et al. (2014)	Low risk	Yes	3048	2168 knees neutrally aligned (90°), 880 varus (<90°)	Varus knees associated with higher revision rate p < 0.0001 No revisions in the neutral group. 30 in the varus group.
Magnussen et al. (2011)	Low risk	No	553	35 knees in varus514 knees neutralFour knees in valgus alignment	All *revisions occurred in neutral* aligned group
Femoral angle malalignment (cFa)					
Ritter et al. (2011)	High risk	Yes	6079	Neutral defined as any angle ≥8 valgus. 91.6 % neutral	7.8 % failure rate associated with valgus malalignment *p* *=* *0.0082*
Kim et al. (2014)	Low risk	Yes	3048	2858 knees alignment was 2.0–8.0° valgus (neutrally aligned group), in 160 knees the alignment was <2.0° valgus (varus aligned group), and in 58 knees the alignment was >8.0° valgus (valgus aligned group)	*30 revisions overall*. 5 % revision rate in varus group *(p* *=* *0.001)* and 1.7 % revision rate in valgus group *(p* *=* *0.1005)*
Magnussen et al. (2011)	Low risk	No	553	24 knees in varus513 knees neutral16 knees in valgus alignment	All *revisions occurred in neutral* aligned group
Tibio-femoral anatomical angle malalignment (cTFaA)					
Berend et al. (2004)	High risk	Yes	3152	*cTFaA—*Mean 3.6° valgus for entire cohort. Mean 1.4° valgus for failure group	Varus tibial component alignment >3° (Hazard Ratio 17.2, p < 0.0001) associated with tibial implant failureOverall varus limb alignment associated with failure
Fang et al. (2009)	High risk	Yes	6277	Mean postoperative *cTFaA was 4.8° (±* *2.5) valgus*. 69 % were in normal alignment (within 1SD of mean)	The revision rate for the neutral alignment group was significantly lower at 0.5 % (21/4029), compared to 1.8 % (18/1222) for the varus group (p = 0.0017) and 1.5 % (12/819) for the valgus group (p = 0.0028)
Ritter et al. (2011)	High risk	Yes	6079	Neutral defined as 2.5–7.471 % neutral	8.7 % failure rate when tibial component <90° and femoral component ≥8° valgus p < 0.0001
Kim et al. (2014)	Low risk	Yes	3048	1928 neutrally aligned (3–7.5° valgus), 664 varus aligned (<3° valgus) and 456 valgus aligned (>7.5°)	*30 revisions overall*. 2.3 % revision rate in varus group *(p* *=* *0.005)* and 0.9 % revision rate in valgus group *(p* *=* *0.91)*
Morgan et al. (2008)	Low risk	No	197	73 neutral (4–9° valgus)58 valgus (>9°)66 varus (<4.9°)	Six revisions overall. *No significant difference between groups (p* *=* *0.78)*

Only one study (Kim et al. 2014) reported on the association between sagittal and axial malalignment demonstrating a significant association between malalignment on these planes and increase revision rates. Details of sagittal and axial malalignment and revision rates for each measure in all studies is presented in (Tables 5 and 6) respectively.

**Table 5:** Studies investigating the association between implants’ sagittal malalignment and revision rates (sTA, sFA)

Author	RAQ criteria for radiological bias	Association between malalignment and worse outcome	Sample size	Alignment data	Findings
Kim et al. (2014)	Low risk	Yes	3048	*Femoral component* 1735 neutrally aligned group (0–3°), 748 were classed as the flexion group(>3° flexion) and 565 classed as extension group(>1° extension)	No revisions in neutral group. 25 (3.3 %) revisions in the flexion group (p = 0.0029). 5 (0.9 %) revisions in the extension group (p = 0.2)
				*Tibial component* 2465 in the neutrally aligned group (0–7°), 553 in the abnormally aligned group (<0 and >7°)	Five revisions in neutrally aligned group. 25 revisions in the abnormally aligned group (p < 0.0001)

**Table 6:** Studies investigating the association between implants’ axial malalignment and revision rates (aTRA, aFRA, aTFMA/aTFCA)

Author	RAQ criteria for radiological bias	Association between malalignment and worse outcome	Sample size	Alignment data	Findings
Kim et al. (2014)	Low risk	Yes	3048	*Rotational alignment of femoral component*: 2490 the rotational alignment of the femoral component was 2–5° external rotation, in 401 knees the rotational alignment was <2° external rotation, and in 157 knees the external rotation was >5°	No revisions required for the 2–5° external rotation group. 27 revisions in the <2° external rotation group (p < 0.0001) and 3 revisions in the >5° group (p = 0.029)
				*Rotational alignment of tibial component*: 2490 the rotational alignment of the femoral component was 2–5° external rotation, in 413 knees the rotational alignment was <2° external rotation, and in 145 knees the external rotation was >5°	1 revision required for the 2–5° external rotation group. 27 revisions in the <2° external rotation group (p < 0.0001) and 2 revisions in the >5° group (p = 0.034)

When studies were examined by the quality of radiological assessment method only one study (Kim et al. 2014) out of five that were deemed low risk of radiological assessment bias reported an association between malalignment and increased revision.

## Discussion

The most interesting finding from this work is that studies measuring malalignment using mechanical axes did not demonstrate an association between malalignment and increased revision rates, while studies using anatomical axes did. One explanation is that anatomical axes are less valid in assessing malalignment, a conclusion supported by the fact that these studies were identified as high risk of radiological assessment bias on the RAQ checklist. However, when viewed from the kinematic perspective, it is entirely possible that a mechanically aligned, but anatomically malaligned implanted prosthesis could fail to recreate a patient’s preoperative kinematics and therefore correlate with worse revision rates. As a result, in a mechanical aligned TKR, if the mechanical axis is not 180° that would be a technical error. Where in kinematic aligned TKA, alignment outside 180° could be intentional to restore patient own anatomy. So the findings which demonstrate that malaligned TKR does not affect survivorship can not be translated to the expected results of kinematic TKA. To the authors’ knowledge this is the first independent review to demonstrate this finding.

We found that in four of the eight studies (Berend et al. 2004; Fang et al. 2009; Kim et al. 2014; Ritter et al. 2011) included there was a significant association between malalignment and increased revision rates. Although all associations were in the same direction (i.e. worse alignment causing higher revision rates), the strength of this association should be viewed with caution given the statistical and radiological quality analyses of the included studies. To scrutinize the radiological assessment methods we devised the RAQ flow diagram for this review (Fig. 1). When applied, only one of the studies (Kim et al. 2014) at a low risk of bias demonstrated an association between malalignment and increased revision rates. However, other studies were still included in this review in order for a conclusion to be drawn from the available evidence. This evidence highlights the need for further studies to be carried out with radiological assessment that is free from bias.

Differences in the timing of the radiographs in relation to implantation can lead to a type of error analogous to a lead-time bias. (Ritter et al. 2011) retrospectively analysed 9483 patients operated between 1983 and 2006 and found failure most likely to occur with tibial component malalignment. The radiological data used in their analysis were obtained at the time of latest follow-up ranging between 2–22.5 years following surgery. This could affect revision rates as malalignment can occur as a result of implant migration rather than malalignment at surgery. Variable weight-bearing status and little evidence of rater reliability assessment also add to the potential systematic error. Ritter et al. also acts as a caveat to the methodological quality scoring system. Although judged to be at low risk they demonstrated a near 50 % loss to follow up.

It was not possible neither deemed beneficial to perform a meta-analysis. The parameters of malalignment were poorly defined for the studies included. Studies presented malalignment data either in terms of deviation from the leg axis in the arithmetic mean or as groups of ‘Aligned’ vs. ‘Malaligned’ or ‘Outliers’. A number of studies restricted their analysis to one or two parameters of alignment. This approach is problematic given the relative interconnection between the alignment components in a TKR. (Berend et al. 2004) found the effect of malalignment in one implant moderated by the alignment of the other. (Ritter et al. 2011) concluded that “Correction” of the alignment of the second component in order to produce an overall neutrally aligned knee replacement when the first component has been malaligned may increase the risk of failure. These findings suggest a complex interplay between all measures of alignment in both the tibial and the femoral components that cannot be simplified to conventional definitions of “malaligned” or “aligned”. Seven of the eight studies included looked solely at the coronal view, only one (Kim et al. 2014) looked at alignment in different planes. Following the findings mentioned above by (Berend et al. 2004; Ritter et al. 2011 and Kim et al. 2014) we believe it is of paramount importance to include all parameters of alignment. Therefore, there is a need for standardisation of terminology and an acknowledgement that malalignment occurs with six degrees of freedom.

A number of studies had relatively small sample sizes, predisposing to type II error; e.g. (Morgan et al. 2008) included only six revisions. The non-significant associations obtained may be due to the small variation in the alignments identified in the sample. It is notable that all studies that had larger sample sizes (over 1000 patients) rated highly with the quality assessment score and found an association between some measure of malalignment and outcome. However, this may be due to heterogeneity in measurements, study designs producing significant systematic error that obscures any association (only one of these studies was low risk of assessment bias using the RAQ score), but there is also likely to be a publication bias. The low number of eligible studies here precludes a formal analysis of this, but it is quite likely that there are a number of unpublished studies with no significant associations found. Indeed, several of the included studies did not specifically report a lack of association in some of their measured parameters.

An additional factor that clouds the issue of sample size is the different study methodologies used. Some large cohort studies have been included with thousands of patients; however, as revision is a rare event, the power of these studies can be limited. Contrasted with case control studies the overall number of revisions included can be high, but the sample size much smaller than the corresponding cohort studies. We recognise that this makes comparisons between studies based on sample size hard to make and so we have reported the number of revisions included in each study where available to compensate for the different study designs as well as reporting the number of studies demonstrating correlations.

The main strength of this review was the systematic fashion it was conducted with and the adherence with the guidelines published by the major research groups, such as the AHRQ. These guidelines included a published research protocol with a clear research question, a broad and comprehensive literature search, an explicit inclusion and exclusion criteria for identifying relevant studies, and a quality control assessment of all the results. Limitations of our review strategy included a search confined to English language. We restricted our search to studies published on or after 2000 which means that more modern implant designs were likely to have been used.

## Conclusion

The finding that only the larger studies in this review found an association raises the question of how important a factor malalignment is when studies of such size are required to demonstrate an association. It may be that malalignment is correlated with outcome but the correlation is small and of dubious clinical significance. On the evidence of this review it is impossible to offer any relative risk of failure compared to malalignment given the problems outlined in measuring alignment, variation of study designs and variation is radiological assessment techniques. This type of information could be gleaned by linking standardised radiological assessment to large databases such as the national joint registries.

A further implication of this study is that mechanical alignment in the coronal plane demonstrated no association with revision rates. This questions the premise of patient specific instrumentation devices based on the mechanically aligned knee when considering revision as the endpoint. Although there is a current trend in the industry towards this, perhaps we should be aiming more towards recreating patients’ original anatomy if revision as an endpoint is to be avoided.
